# Emotional adjustment and peer relationships: The role of behavioural reputation and classroom social climate

**DOI:** 10.1371/journal.pone.0332297

**Published:** 2025-09-18

**Authors:** Robin Banerjee, Mark Wright, Federica Bianco

**Affiliations:** 1 School of Psychology, University of Sussex, Brighton, United Kingdom; 2 School of Humanities and Social Science, University of Brighton, Brighton, United Kingdom; 3 Department of Human and Social Sciences, University of Bergamo, Bergamo, Italy; University of Cambridge, UNITED KINGDOM OF GREAT BRITAIN AND NORTHERN IRELAND

## Abstract

Internalising and externalising difficulties are linked with poor peer relationships, but there are gaps in our understanding of the reputational and contextual factors that connect emotional adjustment, social behaviour, and social acceptance in the peer group. A sample of 1439 children aged 7–13 years from 60 classrooms provided self-report and peer-nomination data at two time points over one school year. Children completed a self-report measure of anger, anxiety, and depressive symptoms, alongside ratings of positive and negative peer experiences. They also provided responses to a sociometric survey, which generated scores of social preference as well as behavioural reputation for cooperative, disruptive, aggressive, shy, and leadership qualities. Anger predicted lower social preference via a less cooperative reputation and a more disruptive reputation. Results also showed that anger had a weak connection with greater social preference via a less shy reputation. Anxiety predicted lower social preference via a more shy reputation. Moreover, a more problematic classroom social climate, in terms of peer experiences, was found to attenuate the negative peer consequences of the disruptive reputation associated with anger. Results clarify the value of locating internalising and externalising difficulties in the peer context. They advance theoretical conceptions of the social consequences of emotional maladjustment, and indicate the relevance of classroom-level social factors in peer socialisation processes.

## Introduction

Children’s levels of social acceptance and their behavioural reputation amongst their peers have long received attention in developmental psychology because difficulties in these areas are intertwined risk factors for later psychological problems [1,2]. We also know from a large and established body of previous work that high levels of negative emotions may be indicative of internalising and externalising psychopathologies, which are associated with lower acceptance within the peer group [3,4]. However, the details of these associations have not been adequately elucidated in longitudinal research, and we are far from a complete understanding of the mechanisms involved. The present study evaluates hypotheses about differentiated associations between emotional adjustment and peer relationships, with attention to the role played by behavioural reputation and the moderating role of the classroom social climate. We sought to shed new light specifically on the pathways that link children’s emotional adjustment to later peer acceptance [5,6] via their behavioural reputation [7].

In this way, the present research was designed to extend theoretical insights into a social-ecological systems approach to children’s development at school, taking the existing conceptualisation of the complex social system of schooling in teaching and learning [8] into an analysis of emotional, behavioural, and socio-relational experience. Of particular theoretical interest is a consideration of how individual pupils’ emotional experience is associated with their peer groups’ collective views on their behavioural reputation, and an analysis of if and how that pattern of associations may be moderated by variations in the wider social climate of the classroom as a whole.

### Emotional adjustment and peer relationships

Common internalising psychopathologies such as anxiety and depression are typically associated with feelings of worry and fear and with feelings of sadness, respectively [9]. In the case of externalising difficulties, core symptoms of aggressive-disruptive and oppositional behaviour are frequently associated with feelings of anger [10]. Difficulty in managing these negative emotions is understood to compromise children’s adaptive functioning in everyday life [11]. One highly relevant aspect of this everyday functioning concerns children’s peer relationships. Despite a broad consensus on the critical role of emotions in social skills [12,13], relatively little research has studied the emotional predictors of peer status, particularly in periods of life beyond early childhood. And while a substantial body of evidence links internalising and externalising difficulties with peer problems in middle childhood and preadolescence [14–16], we still know relatively little about how the negative emotions associated with internalising and externalising difficulties map onto the behavioural reputation of children, and the extent to which they are accepted within their peer group. Such knowledge would have important implications for intervention and prevention efforts.

A significant body of research has demonstrated that peer acceptance is undermined by a reputation for socially withdrawn behaviour or aggressive-disruptive behaviour, or indeed an absence of positive social behaviour characterised by cooperative and leadership qualities [17,18]. Crucially, theoretical models suggest that these behavioural profiles can be tracked back to variations in children’s experience of negative emotions. Functionalist approaches to emotion locate feelings within social context, representing feelings as signals related to the accomplishment of social tasks and goals [19], whether these involve a desire to escape danger or avoid threat (e.g., anxiety), a response to frustration in achieving a goal (e.g., anger), or a consequence of having to relinquish a goal (e.g., sadness).

Thus, we would expect that anger would frequently manifest as starting fights or disrupting group interactions, whereas both worries/fears and sadness would frequently manifest as social withdrawal. In both cases, cooperative and leadership behaviour would seem less likely manifestations of the negative emotion, but some degree of differentiation may be evident here: leadership qualities would be difficult to exhibit in the context of socially withdrawn behaviour, and cooperative behaviour would not sit easily alongside disruptive behaviour [20]. In sum, we propose that a peer reputation for certain profiles of behaviour can explain the link between the negative emotional experiences and lower levels of peer acceptance [21].

### Moderation by classroom social climate

Accumulating evidence shows the association between the climate of a school and children and young people’s mental health [22,23]. One of the highly relevant dimensions of this association concerns the social climate of a given peer group, as this may play a very significant role in determining what kind of peer reputation translates into higher or lower peer acceptance. Preliminary evidence for such a role of the peer context comes from studies on the effects of social withdrawal [24] as well as bullying and aggression in relation to popularity [25,26].

In the present study we focused on the social climate of a classroom in terms of the aggregated level of prosocial or victimising behaviour reported by the children in the class. This kind of social climate of a classroom has already been identified as an important moderator of socio-demographic effects on children’s emotional problems and social competence [27]. Extending this ecological analysis of children’s social development, we propose that the translation of internalising and externalising profiles into relationship outcomes is similarly dependent on the social climate of the classroom.

In particular, we suggest that the negative link between internalising characteristics and peer acceptance is likely to be buffered in a more positive classroom in terms of peer experience (where there is a greater concentration of prosocial and lower concentration of victimising behaviours); the greater prosociality and sensitivity of the social group should serve a protective function for those with internalising difficulties. Analysis at a dyadic level has demonstrated that positive friendships attenuated the link between internalising behaviours and victimising peer outcomes [28], but the effect of a positive classroom climate, reported directly by the children themselves, in attenuating the negative link between internalising behaviours and peer acceptance has not been adequately addressed.

We expect the opposite pattern for the link between externalising behaviours and peer outcomes. Classroom social climate can be conceptualised as setting normative expectations for social behaviour, so classrooms characterised by more prosocial and less victimising behaviour should be intolerant of aggressive and disruptive behaviour and therefore strengthen the negative link between externalising behaviours and peer acceptance [29]. Where the concentration of positive social interactions is at a relatively low starting point, however, we could expect the persistence and growth of externalising problems within social groups [30].

The investigation described here would provide data in the UK context on such patterns, previously studied in the USA [24], China [31], and the Netherlands [32]. Furthermore, it adds to the peer report data of children’s own self-reported emotional experiences and perceptions of social life. Crucially, it also uses a longitudinal design to test for the first time the hypothesis that this moderation effect involving children’s peer groups can account for the way in which specific patterns of emotional adjustment, reported by the children themselves, translate into meaningful relational outcomes.

### Hypotheses

We hypothesised that differentiated pathways linking anger, anxiety, and depression with subsequent peer acceptance in the peer group would be explained by concurrent behavioural reputation. Specifically, anger should be associated with more negative peer relations via a reputation for less cooperative, more disruptive, and more aggressive behaviour, whereas anxious and depressive emotional symptoms should predict less peer acceptance via a reputation for more shy and less leadership behaviour. We also tested the hypothesis that the internalising profile of shy behaviour would be more tolerated (i.e., less likely to predict lower peer acceptance) in classroom environments where the relative frequency of positive peer interactions is higher. In contrast, disruptive and aggressive behaviour were expected to be more tolerated in classrooms with a higher rate of negative reported peer experiences.

We focused on the developmental period from middle childhood through to early adolescence, given that peer group acceptance is a major social goal at this time [33] and that this is a potential window of vulnerability ahead of an epidemiological rise in clinically significant social and emotional difficulties [34]. In addition, given that both emotional characteristics and social behaviour have been closely linked to children’s cognitive appraisals of themselves [35], we controlled for judgements of self-worth across various domains. We also examined whether the pattern of results differed for boys and girls, given the well-known gender difference in both the emotional and the relational domain [36,37].

## Method

### Participants

The sample was drawn from a large population of school children in three local authorities (school districts) that were involved in national English or Welsh government projects on mental health and socio-emotional difficulties. Schools from these local authorities had the opportunity to participate in a programme of longitudinal research on emotional adjustment and peer relations. In total, 94 classes of pupils from 22 primary schools and 4 secondary schools completed the online self-report and sociometric measures described below at the first time point during the first half of the school year, and 60 of those classes completed the measures again at a second time point at the end of the school year. We focus here on the latter 60 classes, comprising 1439 children (50.2% girls) with sociometric data at both time points. The pupils were aged between 7 and 13 years, with the mean and median year group being Year 5 (age 9–10 years). The 60 classes with data at both time points were not significantly different from the 34 classes without follow-up data on any of the self-reported social experience and emotional adjustment measures (all *p* > .05), except that the former scored higher than the latter on negative peer experiences (*p* = .002).

### Measures

#### Emotional adjustment, social experiences, and self-perceptions.

This brief online self-report measure comprised 43 items, including five neutral filler items. There were 7 items on anger (e.g., “I feel angry”), 7 items on depressive symptoms (e.g., “I get so upset that I feel like crying”), and 8 items on anxiety (e.g., “I spend time worrying about things”), rated from 0 (‘Almost never’) to 3 (‘Nearly all the time’). All of the above subscales demonstrated satisfactory internal consistency: anger, *ɑ* = .81; *a*nxiety, *ɑ* = .81; depression, *ɑ* = .82. The items tapped neg*a*tive emotions associated with internalising and externalising difficulties (see Supporting Information, S1 Appendix). Children also completed 5 items on positive/prosocial peer experiences (e.g., “Other pupils say kind things to me”) and 5 items on negative/victimising peer experiences (e.g., “Other pupils say nasty things about me to other people”), taken from an adaptation of the Social Experience Questionnaire [38] to be measured on the same scale as the other items [39]. Cronbach’s alphas were good, .77 ≤ *ɑ* ≤ .84. In addition, children completed a brief measure of evaluative self-perceptions, with 6 items broadly addressing each of the subscales from Self-Perception Profile for Children [40] (e.g., “I like the kind of person I am”, “I am good at schoolwork”) rated from 0 (‘Not at all true’) to 3 (‘Very true’) *ɑ* = .68. Overall mean (SD) scores were 1.90 (.69) for positive peer experiences, .75 (.71) for negative peer experiences, .88 (.62) for anger, .84 (.60) for anxiety, .72 (.63) for depressive symptoms, and 2.10 (.56) for self-worth. The Supporting Information (S1 Appendix) details two pilot studies establishing strong convergence between the anger, anxiety, and depressive subscales and more elaborate existing measures, and a confirmatory factor analysis of the data from the first time point of the present study corroborated the factor structure of the self-reported emotional experiences items, with all items within each subscale loading significantly onto their respective latent factors (standardised coefficients range from .365 to .689; all *p*s < .001) and satisfactory model fit, *Χ*^2^(200) = 1092.23, *p* < .001, CFI = .91, RMSEA = .06, SRMR = .04. Note that covariances were specified within two sets of context-specific items (feeling angry, worried, and sad at home; and feeling angry, worried and sad at school). Measurement invariance across gender was also assessed: configural invariance was indicated by satisfactory model fit, *Χ*^2^(400) = 1370.31, *p* < .001, CFI = .91, RMSEA = .06, SRMR = .04, with no significant deterioration when equal loadings were specified, △*Χ*^2^(19) = 14.13, *p* = .776.

Given that our planned analysis involved use of composite scale scores for emotional adjustment, each child received a mean score across all items within each emotion subscale (possible range 0–3). In addition, a classroom social climate score was created for each class by subtracting the class’s mean score for negative/victimising peer experiences from the class’s mean score for positive/prosocial peer experience scores (possible range −3–3); higher scores thus indicate a greater relative frequency of positive over negative peer experiences. Preliminary analysis showed that the mean across all 60 classrooms was 1.15 (*SD* = .36, range .45 to 1.79), and that the scores were not significantly correlated with year group or with the percentage of girls in the class, *r*s < .13, *p*s > .05. For multigroup analyses to evaluate the moderating effect of classroom social climate, we compared the 20 classrooms (from 14 schools) displaying the highest difference between reported positive and negative peer experiences (i.e., positive experiences were considerably more frequent than negative experiences, *M* = 1.54, *SD* = .12) with the 20 classrooms (from 12 schools) showing the least difference (*M* = .75, *SD* = .18, *t*(38) = 16.16, *p* < .001).

#### Behavioural reputation and social acceptance.

Based on established approaches to sociometric measurement [21], children also completed an online sociometric survey, involving nominations of up to three classmates for 7 items, including brief behavioural descriptors for the reputation items: most like to spend free time with; least like to spend free time with; cooperative (help other people, share, and take turns); disruptive (not very good at being in a group, and don’t listen to other people); shy (really quiet and always seem to be on their own); starts fights (say mean things to other people, or push or hit them); and leader (often seem to be in charge of things, and good at deciding what to do). The total number of nominations received by each child was tallied and standardised within classroom, i.e., converted into z scores with a mean of 0 and SD of 1 for each classroom. The difference in ‘most-like’ and ‘least-like’ nominations received by each child constituted that child’s social preference scores [21].

### Procedure

Following ethics approval from the University of Sussex, School of Life Sciences Research Governance Committee (approvals RB0109 and RB0310), head teachers provided written informed consent to take part in the research. Note that parents received information letters and forms to return if they wished to withdraw their child from participating in the study. Fewer than 5% of participants were withdrawn from the survey administration. In addition, assent was gained from children to complete the two online surveys in their schools’ computer laboratories in 15-minute sessions supervised by school staff. The surveys were completed independently (with audio available for children with literacy needs) and questions were presented one at a time. Children identified themselves and made peer nominations using code numbers. The dataset for our analyses was derived from projects in three UK local authorities where these data were collected in the school year from 1 September 2010–31 July 2011. Surveys were completed once during the first half of the school year (approximately 4 months after the start of the school year), and again at the end of the school year (approximately 6 months after the first time point).

## Results

**Fig 1:**
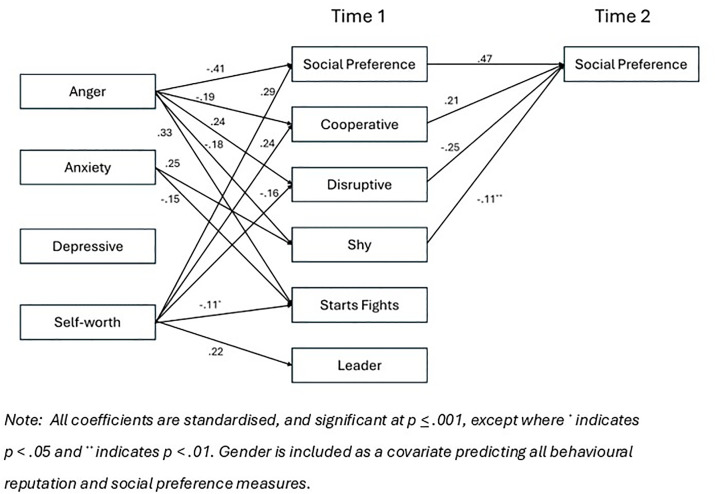
Final model (for entire sample) of associations between emotional adjustment, behavioural reputation, and social preference.

Table 1 shows the inter-correlations for all measures. Using the Lavaan library in R, with Full Information Maximum Likelihood for handling missing data in all analyses, an initial fully saturated structural equation model evaluated pathways from all emotional adjustment and behavioural reputation measures at Time 1 to social preference at Time 2, after controlling for social preference at Time 1. Pathways were also included from all emotional adjustment measures, along with self-worth, to all behavioural reputation measures and social preference at Time 1. Gender was included as a covariate, and covariances were included among all predictor variables. We then trimmed the non-significant paths. The model obtained a good fit, *Χ*^2^(9) = 15.73, *p* = .073, CFI = .998, RMSEA = .023, SRMR = .010, see Fig 1. After controlling for self-worth, anger was positively associated with having a reputation for both disruptive behaviour and starting fights, and negatively predicted a cooperative reputation and a shy reputation. Anxiety was positively associated with a shy reputation, but negatively predicted a reputation for starting fights. Finally, Time 2 social preference was positively predicted by a cooperative reputation and negatively by a shy and a disruptive social reputation.

**Table 1:** Correlations among all variables.

	1	2	3	4	5	6	7	8	9	10
1. Angry	--									
2. Anxious	.58**	--								
3. Depressive	.68**	.76**	--							
4. Self-worth	−.34**	−.26**	−.36**	--						
5. Cooperative	−.19**	−.05*	−.11**	.16**	--					
6. Disruptive	.22**	.04	.11**	−.13**	−.37**	--				
7. Shy	−.05	.15**	.10**	−.05	.14**	−.22**	--			
8. Fight	.21**	−.03	.05	−.09**	−.31**	.75**	−.25**	--		
9. Leadership	−.05	−.06*	−.07**	.13**	.43**	−.10**	−.15**	−.02	--	
10. Soc Pref T1	−.20**	−.14**	−.18**	.16**	.48**	−.47**	−.05	−.32**	.31**	--
11. Soc Pref T2	−.18**	−.13**	−.17**	.13**	.39**	−.39**	−.05	−.29**	.25**	.60**

* *p* < .05 ** *p* < .01. Note. Soc Pref = Social preference. Ns ranged between 1296 and 1439 depending on missing data.

Indirect pathways from Time 1 emotional adjustment to Time 2 social preference, via the relevant Time 1 behavioural reputation scores were estimated, using bootstrapping with 1000 samples. To ensure conservative estimates, the direct paths from Time 1 emotional adjustment to Time 2 social preference were included, even if they did not achieve statistical significance. Anger predicted lower social preference via a less cooperative reputation (standardised estimate = −.04, *p* = .001) and a more disruptive reputation (standardised estimate = −.06, *p* = .001). Anger also modestly predicted greater social preference via a less shy reputation (standardised estimate = .02, *p* = .012). Finally, anxiety predicted lower social preference via a more shy reputation (standardised estimate = −.03, *p* = .006).

**Fig 2:**
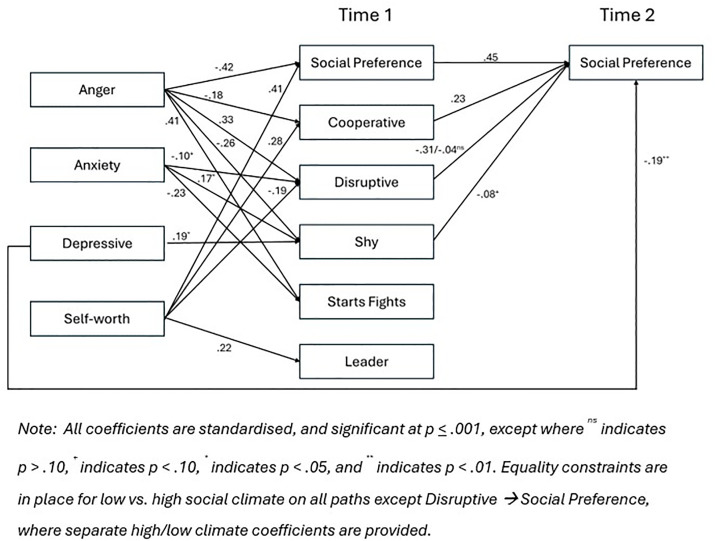
Final multi-group (high and low classroom climate) model of associations between emotional adjustment, behavioural reputation, and social preference.

Our multigroup analysis compared the 20 most and 20 least socially supportive classrooms, as evaluated in terms of peer experiences. Beginning with an initial saturated model, we placed equality constraints on all paths from Time 1 behavioural reputation to Time 2 social preference, after controlling for Time 1 social preference. This showed that constraints do not hold across groups, *Χ*^2^(5) = 15.94, *p* = .007. Inspection of the unconstrained saturated model showed that the negative link from disruptive behaviour to Time 2 social preference was robust in the high social climate classrooms but virtually zero in the low social climate classrooms. When the equality constraint was freed for this path, the chi-square returned to non-significant, *Χ*^2^(4) = 5.27, *p* = .261, ∆*Χ*^2^(1) = 10.67, *p* = .001. The model continued to fit well when paths from emotional adjustment to behavioural reputation were set to be equal across the two classroom social climate groups, *Χ*^2^(33) = 36.97, *p* = .291. The final model, with non-significant paths deleted, had excellent fit, *Χ*^2^(45) = 44.93, *p* = .475, CFI = .1.00, RMSEA = .000, SRMR = .026, see Fig 2. Regarding indirect effects, anger predicted lower social preference via a less cooperative reputation regardless of classroom climate (standardised estimate = −.05, *p* = .002). However, only in classrooms with a higher concentration of positive peer interactions did anger predict lower social preference via more disruptive reputation (standardised estimate = −.12, *p* < .001); the indirect effect in classrooms with a relatively lower concentration of positive peer interactions was non-significant (standardised estimate = −.02, *p* = .579).

It should be noted that the same pattern was found when gender was included as a further grouping variable. Applying equality constraints on all paths from Time 1 behavioural reputation to Time 2 social preference across both low vs. high social climate classrooms and gender showed that constraints do not hold, *Χ*^2^(15) = 29.28, *p* = .015. However, when the equality constraint was freed for the path from disruptive behaviour to Time 2 social preference, only for low vs. high social climate while maintaining equality across gender, goodness of fit was restored, *Χ*^2^(14) = 18.39, *p* = .190, ∆*Χ*^2^(1) = 10.89, *p* = .001.

## Discussion

The current study is aligned with nationwide developments in promoting social and emotional skills and mental health in UK schools [39,41,42]. In line with this, our results shed light on the interplay among emotional adjustment, peer relations and social climate in the classroom, providing information for future tailored intervention. As hypothesised, children’s levels of anger and anxiety had differentiated links with their behavioural reputation within the peer group: reputations were more disruptive and aggressive and less cooperative and shy in the case of those scoring high on anger, whereas reputations were more shy and less aggressive in the case of those scoring high on anxiety, in line with previous literature [43,44]. All of these effects were evident over and above positive pathways between greater self-worth and greater social preference via more cooperative and less disruptive behaviours. However, not all of the reputational characteristics appeared to be socially impactful in terms of predicting changes in social preference over the course of the project.

In particular, the connection between anger and social preference appeared to hinge upon a reputation for more disruptive behaviour and less cooperative behaviour. But surprisingly, an aggressive reputation for starting fights, though more common in those experiencing more anger, was not uniquely predictive of changes in social preference. This perhaps reflects the fact that some aggressive children may be able to establish and maintain positions of social status within peer groups [45]; behaviour that is judged to disrupt social groups, on the other hand, may be more generally problematic and therefore undermine positive social preference [46]. This effect fits with the unexpected positive pathway between anger and peer acceptance via a less shy reputation. Evidently, all other factors being equal, the social engagement that anger may entail may confer some benefits in terms of opening the door to potential social preference (e.g., being proactive about communicating with others). Unfortunately, in the case of children with high levels of anger, the typical pattern of low cooperative and high disruptive behaviour likely makes it difficult for such benefits to materialise [47].

The observed moderating effect of classroom social climate adds a highly relevant set of considerations. Specifically, in classrooms with a lower concentration of positive peer interactions, the predictive link between a disruptive reputation and subsequently lower social preference was nullified. This result adds important longitudinal support to existing evidence that the level of aggression in the classroom moderates the association between students’ aggressive behaviour and social preference, so that as levels of aggression rise in the classroom, the aggressive behaviour of the individual becomes less reliably linked to peer relationship problems [24,31,32].

It could at first glance seem that being in a classroom with more negative peer experiences as reported by pupils could be a protective factor for children with externalising problems, given that it appears to buffer against social rejection, which is widely known to compromise psychological wellbeing [48,49]. However, the present paper did not measure the quality of the relationships that disruptive children had with classmates. Previous literature showed that the friendships of antisocial children are generally not supportive, are short in duration, and feature bossiness and acrimony [50]. Thus, even if socially positive classrooms allow disruptive children to maintain a good social preference, the quality of those interactions may be highly problematic.

In fact, a more significant concern is that in negative classroom ecologies the disruptive behaviour associated with anger evidently can become socially normative and thus tolerable among peers. This could explain the persistence of, and potentially even an increasing concentration of, externalising difficulties within some social groups [30]. This result is thereby relevant to educational and clinical applications, as it speaks to the importance of taking into account the peer context of children when treating their externalising problems, in order to avoid problems escalating to clinical levels. Indeed, in contexts where victimising behaviours are virtually as frequent as prosocial behaviour, children’s aggressive behaviour is likely to be socially reinforced by their peer groups, whose social feedback is that this attitude is a usual, tolerated and successful way of interacting [51].With respect to internalising difficulties, the primary reputational driver for the negative association between anxiety and social preference was the presence of shy or socially withdrawn behaviour. As expected, emotional characteristics that inhibit affiliative behaviour are likely to make it difficult to establish positive relationships with peers [52]. Contrary to the hypotheses, however, a more positive classroom social climate, as reported by pupils, was not found to attenuate the link between a shy reputation and lower social preference. We had expected that a generally more prosocial and less victimising classroom would be more sensitive to, and accepting of, those with an internalising profile [27], but our results underline the significant risks of having a disaffiliative behavioural reputation, even in a classroom that is perceived to be characterised by positive social interactions.

Interestingly, an absence of leadership in anxious children’s reputations did not appear to play a role in their level of social acceptance. Perhaps in a similar way to the absence of clear associations between aggression and social preference, this could underline the potential ambivalence that children have with respect to socially dominant profiles. Those who are seen as leaders and in control of social interactions may not necessarily be preferred interpersonal partners [53].

Contrary to the hypotheses, depressive symptoms did not reveal the same unique pathways to reduced social preference via these reputational characteristics. Although depressive symptoms had significant correlations with various aspects of behavioural reputation as well as with social preference, none of these remained significant once covariation with other negative emotions was included in the model. This suggests that the frequently comorbid negative emotions of anxiety and anger are more likely to drive negative social consequences of depressive symptoms than the sadness and general negativity tapped by the depressive items. This of course does not diminish the significance of the sadness and hopelessness distinctively associated with depressive experiences, but it does suggest that socially-oriented interventions may need to prioritise the management of comorbid anxious and angry emotions that potentially amplify negative self-schemas by undermining successful social interaction and reducing social preference [54].

Finally, it is important to note that gender did not serve as a moderating factor. While this was not a substantive focus of the present work, considerable research in the past has focused on gender differences in relation to children’s emotional profiles in the social world [55]. The fact that the patterns of association among emotional experience, behavioural reputation, and overall social preference were constant across gender – after controlling for gender differences in relation to any given construct – suggests that these fundamental links may be more similar for boys and girls than may first be suspected. However, it may be the case that there are subtle differences in interpretations of behavioural reputation dimensions such as cooperative or disruptive, and this deserves attention in future research.

### Further work

One major direction for future research concerns the study’s restricted window for assessing longitudinal change. Scrutiny of individual trajectories over a longer period will be essential for answering questions about possible bi-directional associations between emotional adjustment, behavioural reputation, and social preference [56]. Also, there is a lack of clarity regarding the causal links. In this regard, a good explanatory candidate might be variations in adaptive and maladaptive forms of emotional self-regulation, relating to skills such as cognitive reappraisal of stressors versus externalising of anger; such aspects of emotion regulation are known to predict social acceptance via behavioural reputation [57]. We believe that future research should include an assessment of this competence, in order to provide a more thorough picture of the interplay between the emotional experience of the child, his/her behavioural reputation, and social acceptance.

A further examination of the antecedents and causal origins of emotional patterns reported by pupils will be also worthy of examination, to better understand reciprocal associations between individual experience and classrooms dynamics. Furthermore, additional research is needed over a longer time frame to elucidate the way in which the observed changes in social preference can reinforce or alter a child’s emotional and behavioural profile. Social information-processing accounts provide a valuable framework for understanding the cognitive processes that might mediate the impact of social experiences on future negative emotions and behavioural responses in subsequent peer interactions [58]. In this kind of extended longitudinal analysis, it may be of particular importance to consider – as we did with gender in the present analysis – the potential moderating role of age, as the patterns of association between emotional experience, behavioural reputation in the peer group, and classroom climate may shift in meaningful ways as children move from early to middle childhood, and then into early and mid-adolescence.

It is important to stress that the present results offer some key insights to incorporate into school interventions, particularly the implementation of programmes and initiatives to promote social and emotional learning. One recent analysis of social, emotional, and mental health needs at school [59] has highlighted the importance of a relational, strengths-based approach to mental health with social and emotional learning at its centre, and the work reported here adds some specificity to this: while interventions may often be targeted at individuals displaying challenging emotional and/or behavioural profiles, our present analyses indicate that consideration of the wider social context needs attention through universal provision, since the social consequences of any given individual characteristic may vary depending on the overall climate of the classroom as a whole.

Indeed, further research is also needed to address the potential moderating roles of wider school (e.g., school climate), community (e.g., neighbourhood deprivation), and broader sociocultural contexts (e.g., ethnicity, cultural values) [27,39,60]. A future exploration of the themes of the present study at the individual, dyadic and group level will also enrich the picture of the reciprocal links between individual emotional functioning and social dynamics in the classroom. Equally, individual differences in temperament, other biological predispositions, and family experiences [61,62] are likely to relate to and interact with the variables in the present analysis. Thus, building on the present findings of differentiated pathways linking anger and anxiety with specific reputational and peer relationship outcomes, a richer understanding of externalising and internalising psychopathology will be gained through more fine-grained analyses of these variables in relation to multiple individual and social-contextual factors.

## Supporting information

S1 Appendix Pilot studies to validate self-report measure of emotional adjustment. (DOCX)
