# Supplementary material for: Emotional adjustment and peer relationships: The role of behavioural reputation and classroom social climate
Source: PLoS One. 2025 Sep 18;20(9):e0332297. doi: 10.1371/journal.pone.0332297 (PMC12445536; doi:10.1371/journal.pone.0332297)
Supplement: S1 Appendix — (DOCX) [file pone.0332297.s001.docx]

**S1 Appendix. Pilot studies to validate self-report measure of emotional adjustment**

Short subscales regarding negative emotions in the My Feelings questionnaire were constructed on the basis of common research descriptions of internalising and externalising psychopathologies, ensuring that they broadly reflected established measures [1-8]. Within each subscale, the items collectively addressed the negative emotions in general terms, at a higher intensity, across different social contexts, and in response to relevant social triggers. Children were asked to rate how often they had the relevant emotional experience on a scale from 0 (almost never) to 3 (nearly all the time).

The anxiety items related to general feelings of anxiety, worry, or fear in different contexts (“I feel worried or afraid”, “I spend time worrying about things”, “I feel worried or afraid when I’m at home”, “I feel worried or afraid when I’m at school”), social fears (“I get nervous about meeting new people”, “I get worried about what other people think of me”), and fears of separation from significant others (“I get worried when I’m away from my parents”), along with a physiological experience of the emotion (“I get so scared that my heart starts to pound”).

The items for depressive symptoms related to general feelings of sadness in different contexts (e.g., “I feel sad”, “I feel sad when I’m at home”, “I feel sad when I’m at school”), overall self-directed negativity (“I think my life is terrible”, “I feel like I hate myself”), feelings of social rejection (“I get upset because no one likes me”), and a physiological experience of the emotion (“I get so upset that I feel like crying”).

The anger items related to general feelings of anger and frustration in different contexts (“I feel angry”, “I feel angry at home”, “I feel angry at school”), varying levels of anger in response to others (“Other people do things that make me really furious”, “I get angry because everyone picks on me”, “People annoy me”), and a persistence of the emotion over time (“When I become angry, I feel angry for a long time”).

In order to provide a preliminary test of convergent and discriminant validity of the online My Feelings questionnaire, one class of 8- to 9-year-olds from each of four schools (34 boys and 47 girls) in one of the participating local authorities completed the questionnaire together with the Child Depression Inventory-Short Form (CDI-S) [5], the Screen for Children Anxiety-Related Emotional Disorders (SCARED) [2], and the Beck Anger Inventory for Youth (BANI-Y) [1]. The total scores for these three measures were each regressed onto all of the My Feelings subscale scores. The only significant predictor of CDI-S was the depressive symptoms subscale (*β* = .60, *p* = .001), the only significant predictor of SCARED was the anxiety subscale (*β* = .61, *p* < .001), and the only significant predictor of BANI-Y was the anger subscale (*β* = .51, *p* = .001).

Further multi-informant validation across time with an at-risk sample was also carried out. A sample of children from 4 secondary and 8 primary schools in the same local authority (N = 290), all of whom had been identified by their school as potentially benefiting from some targeted mental health support, completed the My Feelings questionnaire approximately three months after completing the three measures listed above. Additional ratings from teachers and parents on the Strengths and Difficulties Questionnaire (SDQ; Goodman, 1997) were also obtained for 167 and 48 of those children, respectively. CDI scores were again predicted significantly by the depressive symptoms subscale (*β* = .30, *p* = .002), SCARED was significantly predicted by the anxiety subscale (*β* = .46, *p* < .001), and BANI-Y was significantly predicted by the anger subscale (*β* = .23, *p* = .002). Teacher ratings of emotional problems on the SDQ were significantly predicted by the depressive symptoms subscale (*β* = .38, *p* = .01), whereas teacher ratings of conduct problems were significantly predicted by the anger subscale (*β* = .30, *p* = .011). The statistical power of analyses using the parent ratings was limited given the much smaller sample size, and ratings of emotional problems were not significantly predicted by any of the My Feelings variables. However, parent ratings of conduct problems on the SDQ were significantly predicted by the anger subscale (*β* = .79, *p* = .001).

**References**

1. Beck JS, Beck AT, Jolly JB, Steer RA. *Beck Youth Inventories Second Edition for Children and Adolescents manual.* San Antonio (TX): PsychCorp; 2005.
2. Birmaher B, Khetarpal S, Brent D, Cully M, Balach L, Kaufman J, Neer SM. The Screen for Child Anxiety Related Emotional Disorders (SCARED): Scale construction and psychometric characteristics. *J Am Acad Child Adolesc Psychiatry*. 1997;36(4):545–553.
3. Chorpita BF, Yim L, Moffitt C, Umemoto LA, Francis SE. Assessment of symptoms of DSM-IV anxiety and depression in children: a revised child anxiety and depression scale. *Behav Res Ther.* 2000;38(8):835-55.
4. del Barrio V, Aluja A, Spielberger CD. Anger assessment with the STAXI-CA: psychometric properties of a new instrument for children and adolescents. *Personal. Individ Differ.* 2004;37(2):227-44.
5. Kovacs M. *Children's Depression Inventory: Manual.* North Tonawanda: Multi-Health Systems; 1992.
6. La Greca AM, Stone WL. Social Anxiety Scale for Children-Revised: factor structure and concurrent validity. *J Clin Child Psychol.* 1993;22(1):17-27.
7. Smith RL, Furlong MJ, Bates MP, Laughlin JD. Development of the Multidimensional School Anger Inventory for males. *Psychol Sch.* 1998;35(1):1-15.
8. Spence SH. A measure of anxiety symptoms among children. *Behav Res Ther.* 1998;36(5):545-66.
